# Variations of work engagement and psychological distress based on three working modalities during the COVID-19 pandemic

**DOI:** 10.3389/fpubh.2023.1191314

**Published:** 2023-06-14

**Authors:** Alejandro Unda-López, Clara Paz, Paula Hidalgo-Andrade, Carlos Hermosa-Bosano

**Affiliations:** ^1^Escuela de Psicología y Educación, Universidad de Las Américas, Quito, Ecuador; ^2^Universidad Latina de Costa Rica, San José, Costa Rica

**Keywords:** telework, hybrid work, on-site work, CORE-10, work engagement, COVID-19, UWES-9, psychological distress

## Abstract

With the onset of the COVID-19 pandemic, the rapid spread of the SARS-CoV-2 virus became a global health threat affecting people’s mental and physical health, as well as working conditions and modalities. The reorganization of the work environment also affected work engagement and psychological distress levels. This manuscript assesses how work engagement and distress vary according to gender and age across three working modalities. We used a voluntary response sampling strategy to collect data on psychological distress and work engagement between August 2021 and January 2022. Results are from 542 people working in Ecuador during the COVID-19 pandemic. Overall, participants experienced psychological distress; women and younger participants presented higher psychological distress. Regarding engagement, the sample showed average levels of total engagement, average levels of vigor, and high levels of dedication and absorption. Men presented higher levels of total work engagement and vigor. Psychological distress was significantly and negatively correlated with total work engagement scores and its three factors. There were no differences in work engagement according to the different modalities. However, teleworkers reported significantly higher levels of psychological distress than hybrid workers. Findings are discussed considering ideas for decision-makers to explore the benefits of flexible working practices.

## Introduction

The COVID-19 pandemic and the rapid spread of the SARS-CoV-2 virus became a global health threat affecting people’s mental and physical health (1). The need to mitigate this crisis led to the implementation of preventive measures, including the adoption of new work conditions and modalities. This scenario has further worsened the pandemic’s negative consequences on people’s mental health (1). Due to these changes, the levels of workers’ psychological distress and work-related stress increased, while work engagement and job satisfaction decreased (2, 3), therefore creating the need for measures that promote employee well-being and psychological support in the workplace.

The reorganization of the work environment has also affected work engagement, defined as a positive work-related state of mind characterized by three variables: vigor, dedication, and absorption (4). Vigor refers to a high level of activation and mental resilience regarding work-related tasks, dedication describes a high level of involvement and a sense of enthusiasm and significance, whereas absorption involves high levels of concentration and engrossment in one’s work making it difficult for a person to detach from one’s work (4).

During the COVID-19 pandemic, studies have found that women experience lower levels of vigor, lower job satisfaction, and higher levels of stress at work (5). Additionally, employees who worked from home reported higher levels of work engagement than workers in other modalities due to an increase in sleep hours, effective interactions with supervisors, and avoiding working long hours (6). The variables that predicted work engagement levels were workload, personal resources such as psychological resilience, social support, self-fulfillment (7), available information, available work resources, perception of health (8), work autonomy, modality convenience, and work environment safety (9).

Work engagement and psychological distress are factors usually intertwined in the workplace, contributing to decreased productivity, well-being, job satisfaction, and increased levels of burnout (2, 10). Several factors, such as extended quarantine measures, isolation, infection fear, financial loss, disinformation, and inadequate healthcare, had a significant impact on psychological health (11), resulting in increasing levels of distress, depression, and anxiety in 29.6%, 31,9, and 33.7%, respectively, on a global scale (12). Studies have also found higher levels of psychological distress because of the COVID-19 pandemic. Findings show increased levels of distress among women, younger adults, individuals with lower education, lower income, unemployment, residing in rural areas, and a higher risk of COVID-19 infection (1). Levels of distress among workers were also affected by the work environment (5), living with a partner, living with children under 16, perception of health (13), and physical activity (14). These findings suggest the need for a deeper understanding on how these two variables relate, to create effective interventions that mitigate the outcomes of the pandemic on the workforce.

Previous research has examined the relationships between psychological distress and work engagement and highlighted the existence of both a linear and curvilinear relationship between these constructs (3). Overall findings suggest that psychological distress has a negative correlation with levels of work engagement. However, these correlations tend to vary over time, as work engagement could have a positive effect on mental health in the long term (3). In Ecuador, several studies have analyzed how these variables have affected workers during the COVID-19 lockdown. These studies have shown that higher levels of psychological distress correlate with lower levels of work engagement (5, 15). Further studying this relationship is important as work engagement has been previously associated with better physical (e.g., healthy autonomic cardiovascular activity, better cortisol suppression, better sleep quality) and mental health outcomes (e.g., lower levels of depression, anxiety, and psychological distress) (3, 10).

A deeper understanding of work engagement and psychological distress is needed as it could potentially mitigate negative consequences brought by the pandemic. Moreover, there is limited literature on how these two variables relate to different work modalities (i.e., on-site, telework, and hybrid). Thus, we conducted the present study to assess how work engagement and distress vary according to gender and age, and across three working modalities. We also explored how these variables relate to each other, based on a sample of 542 Ecuadorians working during the COVID-19 pandemic.

## Method

### Design

This study is part of a larger initiative, the main objective of which was to explore productivity and its relationship with the emotional well-being of people who were actively working during the COVID-19 pandemic in Ecuador. The main study used a sequential mixed-methods approach in which quantitative data was first collected, followed by a qualitative phase. The quantitative portion of the study was cross-sectional and non-experimental in nature.

### Participants

We used a voluntary response sampling strategy, primarily through social networks and by contacting twenty medium and large organizations’ Human Resources Departments. To participate in the study, individuals had to be 18 years or older, living in Ecuador, and working at the time. Participants could be either self-employed or salaried employees in either the public or private sector, and working in any modality, including on-site, telework, or hybrid. We collected data between August 2021 and January 2022, a period during which lockdown measures were more flexible, and the majority of the Ecuadorian population had been vaccinated against COVID-19. However, some sectors still employed online and hybrid work modalities.

A total of 542 individuals completed the survey, including 312 women, 229 men, and 1 person who identified as non-binary. The mean age was 35.7 years (SD = 10.35). Of the participants, 257 worked in an on-site modality (47.41%), 119 teleworked (21.95%), and 166 worked in a hybrid modality (30.62%). Most participants were highly educated (86.2%, *n* = 467) and living in urban areas (92.8%, *n* = 503). In terms of their work conditions, 65.9% (*n* = 357) were exclusively working, while 34.1% (*n* = 185) were working and studying at the time of the study.

### Instruments

The data was collected using an online survey available on Microsoft Forms. This article will analyze the following sections:

#### Sociodemographic information

The first section of the questionnaire included multiple choice questions to assess participants’ gender, age, area of current habitation, level of education, current relationship status, whether people had children, number of persons living in their same household, and their current occupation.

#### Clinical Outcomes in Routine Evaluation-10

This is a brief 10-item self-report measure to screen the general level of psychological distress. It includes items regarding depression, anxiety, physical problems, trauma, general functioning, and risk. This instrument uses a Likert-scale format whose response options range from 0 = not at all to 4 = most or all of the time. The total score can be obtained by adding the scores given to each item after reversing items 2 and 3. According to Barkham et al. (16), scores above 1.1 indicate the presence of psychological distress, they also demonstrated that Clinical Outcomes in Routine Evaluation-10 (CORE-10) presented good psychometric properties, indicating a Cronbach Alpha of 0.90. In this study, the Cronbach alpha was 0.85.

#### Utrecht Work Engagement Scale-9

We used this 9-item instrument to assess participants’ level of engagement. Participants responded using a 6-point Likert scale varying from 0 = never to 6 = always. Higher scores indicate higher levels of engagement. A total score can be obtained by adding the scores given to each item. In addition, the items can be grouped into three factors with three items each: vigor (VI), dedication (DE), and absorption (AB). International studies have shown excellent psychometric properties of the 9-item version of the scale, comparable to the 17-item original version (4). In this study, the Cronbach Alpha of the total scale was 0.93. The internal consistency levels of each factor were 0.83 for the VI scale, 0.89 for the DE scale, and 0.77 for the AB scale.

### Procedure

We contacted human resources managers from medium and large companies across various sectors and asked them to complete a survey and distribute it to their colleagues and employees. We further distributed the survey to different social media platforms (e.g., Facebook, Instagram, WhatsApp, and Twitter) and university mailing lists. To access the survey, participants had to review and give their consent to the study. The questionnaire took approximately 15 to 20 min to complete. This study was reviewed by the Ethics Committee of Pontificia Universidad Católica del Ecuador (CEI-09-2021 of 13 August 2021).

### Data analysis

Mean, standard deviation and median were calculated for work engagement, and psychological distress based on work modality. In addition, we analyzed the relationships between the variables of interest and age; to analyze potential gender effects, we performed an independent *t*-test. A non-binary person was excluded from all analyses that included gender as a variable since it is not possible to make comparisons against one participant. Finally, a series of ANCOVAs were conducted to compare the scores of the variables of interest by working modalities, while controlling by age (continuous variable) and gender. For *post hoc* analysis, we used a Tukey contrast for multiple comparisons of the adjusted means. The statistical significance level was set up at 0.05. All the analyses were carried out using R software, available at https://www.R-project.org/.

## Results

CORE-10 scores were negatively correlated with age (*r* = −0.21) whereas engagement (*r* = 0.17) and its three aspects were positively correlated with age (VI = 0.16, DE = 0.15 and AB = 0.17); these correlations, however, were low. When comparing the scores of the variables of interest by gender, CORE-10 [*t*(539) = −3.31, *p* < 0.001], engagement [*t* (539) = 2.47, *p* = 0.01] and vigor scores [*t* (539) = 2.91, *p* < 0.001] presented statistically significant differences. Men showed lower levels of psychological distress (*M* = 1.07, SD = 0.65) and higher levels of engagement (*M* = 4.71, SD = 1.12), and vigor (*M* = 4.61, SD = 1.19) than women (CORE-10: *M* = 1.27, SD = 0.74; engagement: *M* = 4.47, SD = 1.17; VI: *M* = 4.30, SD = 1.28).

Table 1 shows the correlation matrix of the measures of interest (CORE-10, Utrecht Work Engagement Scale-9 (UWES-9) overall score, and UWES-9 factors scores: VI, DE, and AB). CORE-10 score was negatively and significantly correlated with the total score of overall engagement as well as with its three factors (VI = −0.53, DE = −0.45 and AB = −0.31).

**Table 1:** Correlations with confidence intervals for Clinical Outcomes in Routine Evaluation-10 (CORE-10) and Utrecht Work Engagement Scale-9 (UWES-9) scores.

Variable	1	2	3	4
1. CORE-10				
2. VIGOR (VI)	−0.53^**^ [−0.59, −0.47]			
3. DEDICATION (DE)	−0.45^**^ [−0.51, −0.38]	0.83^**^ [0.80, 0.86]		
4. ABSORPTION (AB)	−0.31^**^ [−0.39, −0.24]	0.69^**^ [0.65, 0.74]	0.77^**^ [0.73, 0.80]	
5. ENGAGEMENT (UWE-9)	−0.47^**^ [−0.53, −0.40]	0.92^**^ [0.90, 0.93]	0.95^**^ [0.94, 0.95]	0.89^**^ [0.87, 0.91]

Values in square brackets indicate the 95% confidence interval for each correlation. ^*^Indicates *p* < 0.05. ^**^Indicates *p* < 0.01.

Table 2 shows descriptive statistics for each of the variables of interest, split by working modality. To test the differences between work modalities we conducted a series of ANCOVA tests to determine whether working modality was associated with each of the interest variables (psychological distress and work engagement), we included age and gender as covariates since they showed a relationship with these variables in previous analyses. First, we tested a model including all the variables and all possible interactions between them, but interactions were not significant. Therefore, we tested a model without interactions, and those are the results presented in Table 3. The results indicate that only the psychological distress score presented significant differences between modalities and distress was significantly related to age and gender in this model. After conducting the Tukey test for multiple comparisons of the adjusted means, hybrid and telework modalities presented significant differences (mean difference of the adjusted means = −0.20, *p* = 0.04), suggesting greater levels of psychological distress in people who teleworked.

**Table 2:** Mean, standard deviation and median of CORE-10 and UWES-9 scores by working modalities.

	On-site *n* = 257 (47.4%)	Telework *n* = 119 (22.0%)	Hybrid *n* = 166 (30.6%)	Overall *N* = 542 (100%)
CORE-10				
Mean (SD)	1.21 (0.675)	1.31 (0.784)	1.06 (0.698)	1.19 (0.712)
Median [min, max]	1.20 [0, 3.20]	1.20 [0, 3.60]	1.00 [0, 3.20]	1.10 [0, 3.60]
Engagement				
Mean (SD)	4.48 (1.15)	4.53 (1.20)	4.75 (1.11)	4.57 (1.16)
Median [min, max]	4.56 [0.778, 6.00]	4.89 [1.22, 6.00]	5.00 [1.00, 6.00]	4.78 [0.778, 6.00]
Vigor (VI)				
Mean (SD)	4.37 (1.23)	4.33 (1.32)	4.61 (1.21)	4.43 (1.25)
Median [min, max]	4.33 [0.333, 6.00]	4.67 [0.667, 6.00]	5.00 [1.00, 6.00]	4.67 [0.333, 6.00]
Dedication (DE)				
Mean (SD)	4.60 (1.34)	4.77 (1.32)	4.90 (1.22)	4.73 (1.30)
Median [min, max]	5.00 [0.667, 6.00]	5.33 [1.00, 6.00]	5.33 [0.333, 6.00]	5.00 [0.333, 6.00]
Absorption (AB)				
Mean (SD)	4.46 (1.23)	4.50 (1.26)	4.73 (1.16)	4.55 (1.22)
Median [min, max]	4.67 [0, 6.00]	5.00 [1.00, 6.00]	5.00 [1.00, 6.00]	4.67 [0, 6.00]

**Table 3:** Fixed-effects ANOVA results using CORE-10 and UWES-9 scores as the criteria.

	Predictor	Sum of squares	d*f*	Mean square	*F*	*p*	Partial *η*^2^	Partial *η*^2^ 90% CI [LL, UL]
CORE-10	(Intercept)	87.77	1	87.77	186.12	0.000		
	Modality	3.06	2	1.53	3.25	0.040	0.01	[0.00, 0.03]
	Age	10.53	1	10.53	22.33	0.000	0.04	[0.02, 0.07]
	Gender	4.17	1	4.17	8.83	0.003	0.02	[0.00, 0.04]
	Error	252.77	536	0.47				
Engagement	(Intercept)	548.42	1	548.42	427.62	0.000		
	Modality	5.22	2	2.61	2.03	0.132	0.01	[0.00, 0.02]
	Age	18.29	1	18.29	14.26	0.000	0.03	[0.01, 0.05]
	Gender	6.51	1	6.51	5.08	0.025	0.01	[0.00, 0.03]
	Error	687.42	536	1.28				
Vigor (VI)	(Intercept)	527.66	1	527.66	351.23	0.000		
	Modality	5.09	2	2.54	1.70	0.185	0.01	[0.00, 0.02]
	Age	18.26	1	18.26	12.15	0.001	0.02	[0.01, 0.05]
	Gender	10.68	1	10.68	7.11	0.008	0.01	[0.00, 0.03]
	Error	805.24	536	1.50				
Dedication (DE)	(Intercept)	586.05	1	586.05	356.80	0.000		
	Modality	6.82	2	3.41	2.08	0.126	0.01	[0.00, 0.02]
	Age	17.15	1	17.15	10.44	0.001	0.02	[0.00, 0.04]
	Gender	5.29	1	5.29	3.22	0.073	0.01	[0.00, 0.02]
	Error	880.40	536	1.64				
Absorption (AB)	(Intercept)	532.50	1	532.50	368.80	0.000		
	Modality	5.28	2	2.64	1.83	0.162	0.01	[0.00, 0.02]
	Age	19.51	1	19.51	13.51	0.000	0.02	[0.01, 0.05]
	Gender	4.36	1	4.36	3.02	0.083	0.01	[0.00, 0.02]
	Error	773.93	536	1.44				

LL and UL represent the lower-limit and upper-limit of the partial *η*^2^ confidence interval, respectively.

## Discussion

The COVID-19 pandemic altered many aspects of people’s lives, including mental and physical health (1) and work (8). This article aimed to explore the relationship between work engagement and psychological distress across three work modalities in people employed in Ecuador throughout the COVID-19 pandemic.

Overall results indicate that participants experienced psychological distress and support previous findings (17–19) show that younger participants tend to present higher psychological distress. Regarding gender, our results are also coherent with most studies conducted during the pandemic. In our study, men presented significantly lower levels of psychological distress, showing that the toll on psychological well-being has been heavier for females regardless of the countries and professions (17, 18, 20–22). These gender differences might be due to the additional burdens that women faced even before the pandemic and exacerbated during it (e.g., house chores, childcare, and caregiving) and might also relate to the low perceived productivity among women in remote work reported in a systematic review (23). One exception comes from a study in Japan that shows that the impact on psychological distress was higher among men (24).

Work engagement is crucial for the organization and for employees’ well-being. Our sample showed average levels of total engagement, average levels of vigor, and high levels of dedication and absorption. There are mixed results in the literature regarding job engagement’s relationship with age (25). In our sample, work engagement and its three aspects positively correlated with age, and older participants tended to show higher levels of work engagement. Other factors, such as work intensity (26), aging anxiety, and perceived age discrimination (25) should be explored to better understand the age-engagement correlation.

On the other hand, regarding gender and engagement, results indicate that men presented higher levels of total work engagement and one of its factors, vigor. This is consistent with another research conducted during the pandemic (26). We did not find significant differences in work engagement according to working modalities. This differs from previous research which found that teleworkers showed more job engagement than on-site workers (27). It is necessary to explore the possible causes and factors that influence work engagement. For example, as research suggests, given the context of forced telework during the pandemic, high telework intensity (26) and involuntariness in such work modality might play a role in decreasing work engagement and increasing exhaustion (28).

When looking into the relationship between psychological distress and work engagement, our results are in line with previous literature (5) as CORE-10 scores were negatively and significantly correlated with the total work engagement scores as well as its three factors. Studies have also found that work engagement is related to psychological distress in the short-term and related to positive effects on mental health in the long term (3, 5). Using the conservation of resources theory, Shimazu et al. (3) argue that engaged employees will accumulate resources while performing their jobs, and this accumulation is related to increased physical and mental health. We believe our results support these findings.

Each working modality has its advantages and disadvantages. However, it is important to understand how work engagement and psychological distress are present in each modality to create and implement effective human resources plans. Thus, one of the contributions of this study is the comparison of these variables across different work modalities. Our results indicate that on-site workers presented high engagement, average vigor and dedication, and high levels of absorption. Teleworkers reported average engagement and vigor, and high dedication and absorption. Workers in hybrid modalities presented high engagement, average vigor, and high levels of dedication and absorption. However, similar to other research (29), there were no significant differences in work engagement or its three factors. This result differs from a study in Turkey that found a weaker relationship between absorption in work in those working onsite, and a stronger relationship in those working in a hybrid context, followed by the strongest relationship in those working remotely (30).

On the other hand, our results indicate that on-site workers and teleworkers presented distress while workers in hybrid modalities presented no distress. When analyzing the difference in psychological distress levels between modalities; teleworkers reported significantly higher levels of psychological distress than hybrid workers. Supporting these results, studies have found that people telework can expose people to psychosocial risks, such as lack of support and isolation (31) and that on a hybrid modality present several challenges such as increased working hours, decreased productivity, and a lower capacity for time management due to work-unrelated factors (17, 32). However, an adequate adaptation to a remote working modality, flexible working hours, fair working conditions, and a dedicated workspace are factors related to lower levels of psychological distress and higher levels of life-and work-satisfaction (17, 32). Loneliness and isolation are also important variables. Research shows that remote work predicts decreased job engagement only for workers with low and moderate levels of loneliness (33) and that overwork and isolation might explain stress when working from home (31).

This study shows a first approach to understanding work engagement and psychological distress in different work modalities in Ecuador, a country that had mobility restrictions and forced telework for almost 2 years since March 2020. However, some limitations must be acknowledged. As with any other cross-sectional study, it is impossible to make causal inferences about the relationships between variables and examine how these relationships change over time. Given the sampling and data collection techniques used in this study, we were unable to validate self-responses and track non-response rate. Also, the respondents varied in contexts, locations within the country, and industry. We believe our data is susceptible to nonresponse bias and self-selection bias as an internet connection was needed to ensure participation. People with no internet access may live in contexts that may affect their psychological distress and work engagement levels differently. Further research is needed to improve our understanding of the interactions between these variables in different contexts, to understand which working practices in each modality enhance well-being, as well as ways to improve people’s adaptation and training to succeed in those modalities. Also, other factors such as self-efficacy, training transfer, job satisfaction (34), organizational identification (30), social support (35), loneliness or isolation (31, 33), supervision support (36), position of the employees, and employee experience (23) should be included in future research.

In conclusion, this study helps understand the relationships between psychological distress and work engagement considering gender, age, and work modality. Our results suggest that psychological distress correlates negatively with work engagement and age, while engagement correlates positively with age. Also, men showed lower distress and more total engagement and vigor. Results also indicate that, even though psychological distress was present in the overall sample, there were higher levels of it among people who teleworked compared to those in hybrid working modalities. There were no significant differences in engagement by working modalities. These findings should motivate decision-makers to explore the benefits of hybrid modalities and other flexible working practices; challenge the idea that on-site work is better than other work modalities; redesign jobs with a gender perspective; better support teleworkers by considering their well-being by looking for different ways to connect, encourage social interactions, and promote resilience (33); improve supervision and leadership for all workers regardless of their work modality (36).

## Data availability statement

The raw data supporting the conclusions of this article will be made available by the authors, without undue reservation.

## Ethics statement

The studies involving human participants were reviewed and approved by Pontificia Universidad Católica del Ecuador (CEI-09-2021 of 13 August 2021). The patients/participants provided their written informed consent to participate in this study.

## Author contributions

AU-L, CP, and PH-A designed the study, implemented the study, and collected data. CP and CH-B performed data analysis and reported results. All authors contributed to the article and approved the submitted version.

## Funding

This research was funded by Universidad de Las Américas (PSI.PHA.22.01).

## Conflict of interest

The authors declare that the research was conducted in the absence of any commercial or financial relationships that could be construed as a potential conflict of interest.

## Publisher’s note

All claims expressed in this article are solely those of the authors and do not necessarily represent those of their affiliated organizations, or those of the publisher, the editors and the reviewers. Any product that may be evaluated in this article, or claim that may be made by its manufacturer, is not guaranteed or endorsed by the publisher.
